# The stimulatory effect of the octadecaneuropeptide ODN on astroglial antioxidant enzyme systems is mediated through a GPCR

**DOI:** 10.3389/fendo.2012.00138

**Published:** 2012-11-21

**Authors:** Yosra Hamdi, Hadhemi Kaddour, David Vaudry, Salma Douiri, Seyma Bahdoudi, Jérôme Leprince, Hélène Castel, Hubert Vaudry, Mohamed Amri, Marie-Christine Tonon, Olfa Masmoudi-Kouki

**Affiliations:** ^1^Laboratory of Functional Neurophysiology and Pathology, Research Unit UR/11ES09, Department of Biological Sciences, Faculty of Science of Tunis, University Tunis El ManarTunis, Tunisia; ^2^Inserm U982, Laboratory of Neuronal and Neuroendocrine Communication and Differentiation, University of RouenMont-Saint-Aignan, France; ^3^International Associated Laboratory Samuel de ChamplainMont-Saint-Aignan, France; ^4^Regional Platform for Cell Imaging of Haute-Normandie, Institute for Medical Research and Innovation, University of RouenMont-Saint-Aignan, France

**Keywords:** astrocyte, catalase, ODN, ODN metabotropic receptor, oxidative stress, SOD, protein kinase A

## Abstract

Astroglial cells possess an array of cellular defense systems, including superoxide dismutase (SOD) and catalase antioxidant enzymes, to prevent damage caused by oxidative stress on the central nervous system. Astrocytes specifically synthesize and release endozepines, a family of regulatory peptides including the octadecaneuropeptide (ODN). ODN is the ligand of both central-type benzodiazepine receptors (CBR), and an adenylyl cyclase- and phospholipase C-coupled receptor. We have recently shown that ODN is a potent protective agent that prevents hydrogen peroxide (H_2_O_2_)-induced inhibition of SOD and catalase activities and stimulation of cell apoptosis in astrocytes. The purpose of the present study was to investigate the type of receptor involved in ODN-induced inhibition of SOD and catalase in cultured rat astrocytes. We found that ODN induced a rapid stimulation of SOD and catalase gene transcription in a concentration-dependent manner. In addition, 0.1 nM ODN blocked H_2_O_2_-evoked reduction of both mRNA levels and activities of SOD and catalase. Furthermore, the inhibitory actions of ODN on the deleterious effects of H_2_O_2_ on SOD and catalase were abrogated by the metabotropic ODN receptor antagonist cyclo_1-8_[Dleu^5^]OP, but not by the CBR antagonist flumazenil. Finally, the protective action of ODN against H_2_O_2_-evoked inhibition of endogenous antioxidant systems in astrocytes was protein kinase A (PKA)-dependent, but protein kinase C-independent. Taken together, these data demonstrate for the first time that ODN, acting through its metabotropic receptor coupled to the PKA pathway, prevents oxidative stress-induced alteration of antioxidant enzyme expression and activities. The peptide ODN is thus a potential candidate for the development of specific agonists that would selectively mimic its protective activity.

## INTRODUCTION

The octadecaneuropeptide (ODN) is a peptide generated through the proteolytic cleavage of the 86-amino acid precursor diazepam-binding inhibitor (DBI; Guidotti et al., 1983) which is exclusively expressed in astroglial cells in the central nervous system (CNS) of mammals (Malagon et al., 1993; Burgi et al., 1999; Tonon et al., 2006). DBI and its derived peptides are collectively designated by the term endozepines (Tonon et al., 2006). It was initially reported that ODN acts as an inverse agonist of central-type benzodiazepine receptors (CBR) that are intrinsic components of the GABA_A_ receptor-chloride channel complex (Ferrero et al., 1986). It has been subsequently shown that ODN can also activate a G_i/0_ protein-coupled receptor leading to the activation of phospholipase C (PLC) in astrocytes (Patte et al., 1995; Leprince et al., 2001). In addition, recent data indicate that the ODN G protein-coupled receptor can also activate adenylyl cyclase (AC; Hamdi et al., 2012). ODN exerts a wide range of biological activities which are mediated either through CBR, i.e., increase of aggressiveness and anxiety (Kavaliers and Hirst, 1986; De Mateos-Verchere et al., 1998), reduction of pentobarbital-induced sleeping time and drinking (Dong et al., 1999; Manabe et al., 2001), or through a metabotropic receptor, i.e., inhibition of food intake (Do Rego et al., 2007). Similarly, at the cellular level, the diverse effects of ODN are mediated either through CBR, i.e., stimulation of glial cell and neuroblast proliferation (Gandolfo et al., 1999; Alfonso et al., 2012) and activation of neurosteroid biosynthesis (Do Rego et al., 2001), or through a metabotropic receptor, i.e., increase of intracellular calcium concentration in astrocytes (Leprince et al., 2001) and modulation of neuropeptide expression in neurons (Compère et al., 2003, 2004).

Oxidative stress, resulting from excessive production of reactive oxygen species (ROS), such as hydrogen peroxide (H_2_O_2_), is implicated in the pathology of several neurological disorders including cerebral ischemia and neurodegenerative diseases (Garcia et al., 2012; Hayashi et al., 2012). An excess of H_2_O_2_ induces imbalance in ROS generation, impairs cellular antioxidant defences and finally triggers cell death by apoptosis (Emerit et al., 2004; Shibata and Kobayashi, 2008). It is well known that astroglial cells contain high levels of ROS scavenger molecules such as glutathione (Dringen et al., 1999) and the antioxidant enzymes Mn- and Cu,Zn-superoxide dismutases (Mn- and Cu,Zn-SOD), catalase and glutathione peroxidase (Lindenau et al., 2000; Sokolova et al., 2001; Saha and Pahan, 2007). Nonetheless, astroglial cells can be affected, in terms of viability and functionality, by an insurmountable oxidative stress (Ferrero-Gutierrez et al., 2008; Park et al., 2009). In particular, it has been shown that inhibition of SOD and/or catalase activities in cultured astrocytes is associated with an exacerbation of oxidative damages induced by H_2_O_2_ or hypoxia (Desagher et al., 1996; Bi et al., 2008; Li et al., 2008). Reciprocally, cultured astrocytes derived from Cu,Zn-SOD-overexpressing transgenic mice exhibit increased resistance to oxidative stress (Chen et al., 2001; Wang et al., 2005). However, little is known regarding the endogenous factors that modulate glial antioxidant systems. In this context, we have previously reported that, in cultured astrocytes, ODN exerts a potent protective effect against oxidative stress-induced apoptosis, and attenuates H_2_O_2_-evoked inhibition of SOD and catalase activities (Hamdi et al., 2011). More recently, we have shown that the anti-apoptotic activity of ODN is mediated through the metabotropic endozepine receptor (Hamdi et al., 2012). In contrast, regarding the effects of ODN on endogenous antioxidant systems, the receptor and the signaling mechanism are currently unknown. The purpose of the present study was thus to examine the effects of ODN on SOD and catalase gene expression and to determine the type of receptor involved in the antioxidant action of ODN on astroglial cells.

## MATERIALS AND METHODS

### ANIMALS

All experiments were performed in accordance with American Veterinary Medical Association. Approval for these experiments was obtained from the Medical Ethical Committee for the Care and Use of Laboratory Animals of Pasteur Institute of Tunis (approval number: FST/LNFP/Pro 152012).

### REAGENTS

Dulbecco’s modified Eagle’s medium (DMEM), F12 culture me-dium, D(+)-glucose, L-glutamine, *N*-2-hydroxyethylpiperazine-*N*-2-ethane sulfonic acid (HEPES), fetal bovine serum (FBS), the antibiotic-antimycotic solution, and trypsin-EDTA were obtained from Gibco (Invitrogen, Grand Island NY, USA). Bovine liver catalase, chelerythrine, DL-epinephrine, H89, Triton X-100, and insulin were purchased from Sigma Aldrich (St. Louis, MO, USA). Flumazenil was a generous gift from Hoffmann-La Roche (Basel, Switzerland). Rat ODN and the G protein-coupled receptor antagonist cyclo_1-8_[DLeu^5^]OP were synthesized by using the standard Fmoc procedure, as previously described (Leprince et al., 2001). All other reagents were of A grade purity.

### SECONDARY CULTURES OF RAT CORTICAL ASTROCYTES

Secondary cultures of rat cortical astrocytes were prepared as previously described (Castel et al., 2006). Briefly, cerebral hemispheres from newborn Wistar rats were collected in DMEM/F12 (2:1; v/v) culture medium supplemented with 2 mM glutamine, 1% insulin, 5 mM HEPES, 0.4% glucose, and 1% of the antibiotic-antimycotic solution. Dissociated cells were resuspended in culture medium supplemented with 10% FBS, plated in 175-cm^2^ flasks (Greiner Bio-one GmbH, Frickenhausen, Germany), and incubated at 37°C in a 5% CO_2_/95% O_2_ atmosphere. When cultures were confluent, astrocytes were isolated by shaking overnight the flasks with an orbital agitator and plated on 35-mm Petri dishes at a density of 0.3 × 10^6^ cells/ml. All experiments were performed on 5- to 7-day-old secondary cultures.

### QUANTITATIVE RT-PCR ANALYSIS

Cultured cells were incubated at 37°C with fresh serum-free medium. At the end of the incubation, the culture medium was removed and the cells were washed twice with phosphate buffered saline (PBS; 0.1 M, pH 7.4). Total RNA was extracted by using Tri reagent (Sigma, St Quentin Fallavier, France) and purified using the NucleoSpin kit (Macherey-Nagel, Hoerd, France). cDNA was synthetized from 3–4 µg of total RNA with ImProm II Promega kit (Promega). Quantitative RT-PCR was performed on cDNA in the presence of a 1× Fast SYBR Green universal PCR Master mix (Applied Biosystems, Courtaboeuf, France) containing concentrations of dNTPs, MgCl_2_, SYBR green reporter dye, AmpliTaq Gold DNA polymerase, and forward (5′-CCTTCTTGTTCTGCAACC-TGCTA-3′) and reverse (5′-CCGGACTCTCCGGTATCTGA-3′) SOD (GenBank accession no. NM_012880) primers, or forward (5′-CCACAGTCGCTGGAGAGTCA-3′) and reverse (5′-GTTTC-CCACAAGGTCCCAGTT-3′) catalase (GenBank accession no. NM_012520) primers, or forward (5′-CAGCCTCGTCTCATAGA-CAAGATG-3′) and reverse (5′- CAATGTCCACTTTGTCACAAG-AGAA-3′) glyceraldehyde-3-phosphate dehydrogenase (GADPH, GenBank accession no. NM_017008) primers (300 nM, each; Proligo, Paris, France), using the ABI Prism 7000 sequence detection system (Applied Biosystems). The amount of SOD and catalase cDNA in each sample was calculated by the comparative threshold cycle (Ct) method and expressed as 2^-δδCt^ using GADPH as an internal control.

### MEASUREMENT OF ANTIOXIDANT ENZYME ACTIVITIES

Cultured cells were incubated at 37°C with fresh serum-free medium. At the end of the incubation, cells were washed twice with PBS and total cellular proteins were extracted by using the lysis buffer containing 50 mM Tris–HCl (pH 8.0), 10 mM EDTA, 100 µM phenylmethylsulfonyl fluoride, and 1% Triton X-100. The homogenate was centrifuged (16,000 *g*, 4°C, 20 min) and the cellular extract contained in the supernatant was stored at -20°C until enzyme activity determinations.

The activity of SOD was measured using a spectrophotometric assay, which consists in measuring epinephrine autoxidation induced by superoxide anion. Samples, prepared as described above, were incubated for 3 min with a mixture containing bovine catalase (0.4 U/µl), DL-epinephrine (5 mg/ml), and Na_2_CO_3_/NaHCO_3_ buffer (62.5 mM, pH 10.2). The oxidation of epinephrine was measured at 480 nm with a Bio-Rad spectrophotometer (Bio-Rad Laboratories, Philadelphia, PA, USA).

The activity of catalase was determined on the basis of the decrease of H_2_O_2_. Samples, prepared as described above, were mixed with 30 mM H_2_O_2_ in PBS. The disappearance of H_2_O_2_ was measured at 240 nm for 180 s at 30 s intervals. Catalase activity was calculated using the extinction coefficient of 40/mM/cm for H_2_O_2_.

### STATISTICAL ANALYSIS

Data are presented as the mean ± SEM from three independent experiments performed in quadruplicate. Statistical analysis of the data was performed by using Student’s *t*-test, ANOVA, followed by Bonferroni’s test, and two-way ANOVA test. A *p*-value of 0.05 or less was considered as statistically significant.

## RESULTS

### ODN INCREASES SOD AND CATALASE mRNA LEVELS IN CULTURED ASTROCYTES

**FIGURE 1:**
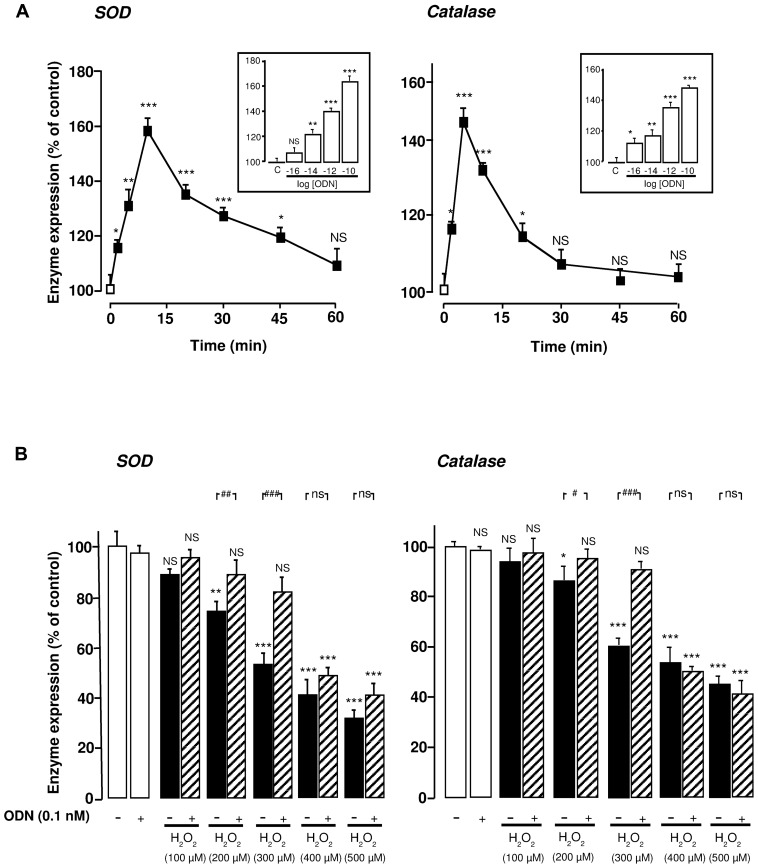
**Protective effects of ODN against H_**2**_O_**2**_-evoked inhibition of SOD and catalase mRNA levels in cultured rat astrocytes**. **(A)** Effects of ODN on SOD and catalase mRNA levels. Astrocytes were incubated in the absence or presence of ODN (0.1 nM) for the times indicated. Inset, cells were treated for 10 min with increasing concentrations of ODN (0.01 pM to 0.1 nM). SOD and catalase mRNA levels were measured by quantitative RT-PCR. Data were corrected using the GAPDH signal as an internal control and the results are expressed as a percentage of controls. Each value is the mean (±SEM) of at least four different wells from three independent experiments. ANOVA followed by the Bonferroni’s test. *p< 0.05; **p < 0.01; ***p < 0.001; NS, not statistically different vs*. *control. **(B)** Effects of ODN on H_2_O_2_-evoked inhibition of SOD and catalase mRNA levels. Cells were pre-incubated for 10 min in the absence or presence of 0.1 nM ODN, and then incubated for 1 h with medium alone or with graded concentrations of H_2_O_2_ (100–500 µM) in the absence or presence of ODN. The results are expressed as a percentage of control. Each value is the mean (±SEM) of at least four different wells from three independent experiments. Analyses similar to those in **(A)** were performed and symbols show the significance vs. H_2_O_2_-treated cells: ^#^*p *< 0.05; ^##^*p* < 0.01; ^###^*p* < 0.001; ns, not statistically different.

We have previously shown that picomolar concentrations of ODN suppress the inhibitory effects of 300 µM H_2_O_2_ on SOD and catalase activities in cultured rat astrocytes (Hamdi et al., 2011). To explore the mechanism involved in the effect of ODN on antioxidant enzyme systems we monitored SOD and catalase gene expression by quantitative PCR. Time-course experiments revealed that ODN (0.1 nM) significantly enhanced SOD and catalase mRNA levels within 2 min with a maximum effect after 10 min and 5 min of incubation, respectively (**Figure 1A**). Thereafter, the stimulatory effect of ODN on SOD and catalase expression gradually declined and vanished 60 and 30 min after the onset of ODN administration, respectively. Exposure of astrocytes to increasing concentrations of ODN (0.01 pM to 0.1 nM) induced a concentration-dependent increase of SOD and catalase mRNA levels (**Figure 1A**, inset). In contrast, incubation of astrocytes with graded concentrations of H_2_O_2_ (100–500 µM) dose-dependently decreased both SOD and catalase mRNA levels (**Figure 1B**). We next examined the effects of ODN/H_2_O_2_ co-incubation on enzyme expression. For moderate concentrations of H_2_O_2_ (100–300 µM), ODN (0.1 nM) restored SOD and catalase mRNA levels above control, whereas for higher concentrations of H_2_O_2_ (400 and 500 µM), ODN only partially prevented the decrease of SOD and catalase gene expression (**Figure 1B**).

### ODN BLOCKS H_2_O_2_-EVOKED INHIBITION OF SOD AND CATALASE mRNA LEVELS AND ACTIVITIES THROUGH ACTIVATION OF A METABOTROPIC RECEPTOR COUPLED TO THE PKA PATHWAY

**FIGURE 2:**
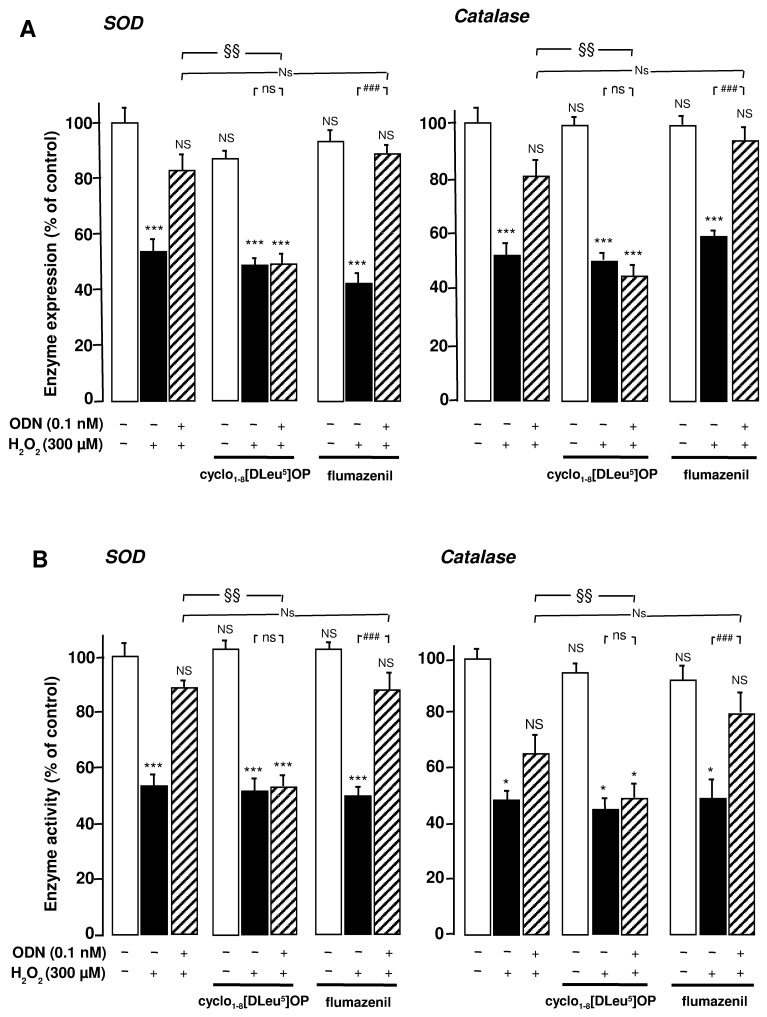
**Involvement of the G protein-coupled receptor of ODN in its protective effects against the deleterious action of H_**2**_O_**2**_ on SOD and catalase in cultured rat astrocytes**. Astrocytes were pre-incubated for 30 min in the absence or presence of the metabotropic receptor antagonist cyclo_1-8_ [Dleu^5^] OP (1 µM) or the CBR antagonist flumazenil (1 µM) and then incubated for 1 h with medium alone or with 300 µM H_2_O_2_ without or with ODN (0.1 nM). **(A)** SOD and catalase mRNA levels were quantified as described in **Figure 1**. The results are expressed as a percentage of control. Each value is the mean (±SEM) of at least three different wells from three independent experiments. **(B)** The activity of SOD was measured using a spectrophotometric assay which consists in measuring epinephrine autoxidation induced by superoxide anion, and catalase activity was determined on the basis of the decomposition of H_2_O_2_. The results are expressed as a percentage of SOD or catalase activity with respect to control. Each value is the mean (±SEM) of at least four different dishes from three independent experiments. ANOVA followed by the Bonferroni’s test: [(**A**, SOD) *F* = 7.69, *df* = 40; (**A**, catalase) *F* = 8.58, *df* = 39; (**B**, SOD) *F* = 13.41, *df* = 44; (**B**, catalase) *F* = 7.58, *df* = 44]; *p < 0.05; ***p < 0.001; NS, not statistically different vs. control. ^###^*p* < 0.001; ns, not statistically different vs. H_2_O_2_-treated cells. Two-way ANOVA test: [(**A**, SOD) *F* = 5, *df* = 17; (**A**, catalase) *F* = 8.13, *df* = 17; (**B**, SOD) *F* = 5.87, *df *= 20; (**B**, catalase) *F* = 2.79, *df* = 19]; ^§^*p* < 0.05; ^§§^*p* < 0.01; Ns, not statistically different vs. ODN + H_2_O_2_-cotreated cells.

We next examined the type of receptor of ODN involved in the stimulatory effects of ODN on endogenous antioxidant systems. Administration of the selective metabotropic receptor antagonist cyclo_1-8_[DLeu^5^]OP (1 µM) to cultured astrocytes did not induce any modification of SOD and catalase mRNA levels and activities, but totally abolished the effects of 0.1 nM ODN on H_2_O_2_-evoked inhibition of antioxidant enzyme gene transcription and activities. In contrast, the CBR antagonist flumazenil (1 µM) did not affect the protective action of ODN against the deleterious effect of H_2_O_2_ on endogenous antioxidant systems (**Figures 2A,B**).

**FIGURE 3:**
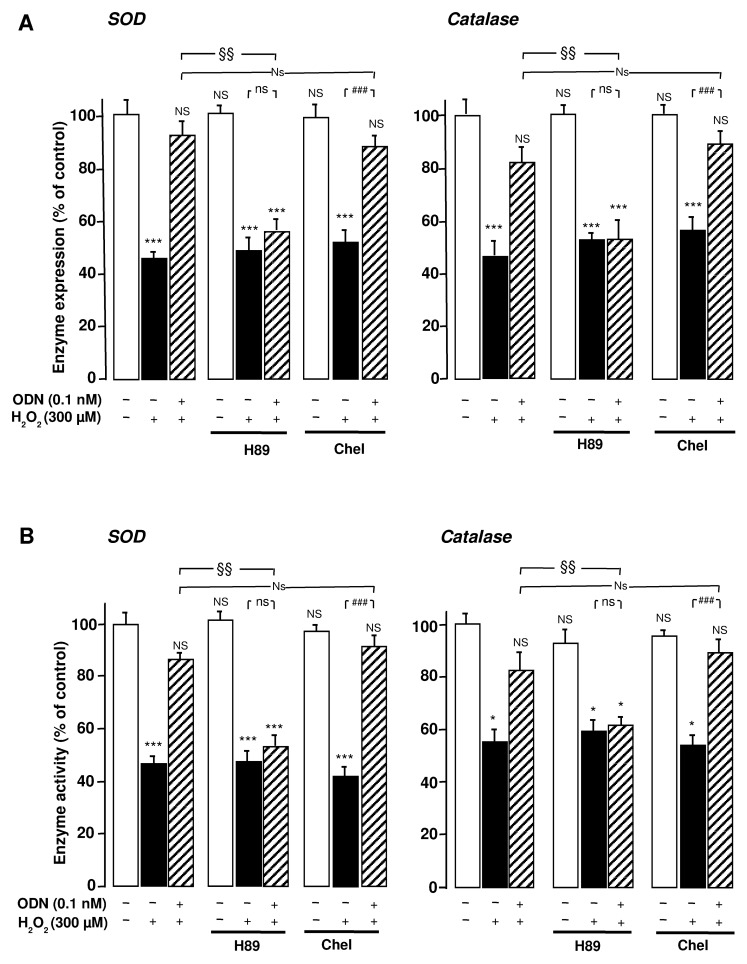
**Protein kinase A-dependence of the protective effects of ODN on the expression and activities of SOD and catalase in cultured rat astrocytes**. Astrocytes were pre-treated for 30 min in the absence or presence of the PKA inhibitor H89 (20 µM) or the PKC inhibitor chelerythrine (Chel; 0.1 µM) and then incubated for 1 h with medium alone or with 300 µM H_2_O_2_ without or with ODN (0.1 nM). **(A)** SOD and catalase mRNA levels were quantified as described in **Figure 1**. The results are expressed as a percentage of controls. Each value is the mean (±SEM) of at least three different wells from three independent experiments. **(B)** The activity of SOD and catalase were quantified as described in **Figure 2**. The results are expressed as a percentage of SOD or catalase activity with respect to control. Each value is the mean (±SEM) of at least four different dishes from three independent experiments. ANOVA followed by the Bonferroni’s test: [(**A**, SOD) *F* = 7.49, *df* = 65; (**A**, catalase) *F* = 5.08, *df* = 53; (**B**, SOD) *F* = 12.37, *df *= 55; (**B**, catalase) *F* = 9.06, *df* = 60]; *p < 0.05; ***p < 0.001; NS, not statistically different vs. control. ^###^*p* < 0.001; ns, not statistically different vs. H_2_O_2_-treated cells. Two-way ANOVA test: [(**A**, SOD) *F* = 2.40, *df* = 20; (**A**, catalase) *F* = 2.63, *df* = 17; (**B**, SOD) *F* = 6.97, *df* = 18; (**B**, catalase) *F* = 4.68, *df* = 24]; ^§^*p* < 0.05; ^§§^*p* < 0.01; Ns, not statistically different vs. ODN + H_2_O_2_-cotreated cells.

Incubation of astrocytes with the selective protein kinase A (PKA) inhibitor H89 (20 µM) abrogated the effect of ODN on the inhibitory action of H_2_O_2_ on SOD and catalase mRNA levels and activities. In contrast, administration of the protein kinase C (PKC) inhibitor chelerythrine (0.1 µM) did not modify the effects of ODN (**Figures 3A,B**), indicating that only the PKA pathway is involved in the protective activity of ODN.

## DISCUSSION

Alteration of endogenous antioxidant systems, especially decrease of SOD and catalase activities, causes exacerbation of oxidative damages leading to apoptosis in various cell types, including astroglial cells (Giffard and Swanson, 2005; Lopez et al., 2007; Bi et al., 2008). Here, we demonstrate that ODN prevents the decrease of SOD and catalase mRNA levels and activities induced by H_2_O_2_ in cultured astrocytes, through activation of a metabotropic receptor positively coupled to the AC/PKA signaling pathway.

We have previously reported that, in cultured astrocytes, ODN at very low concentrations (in the picomolar range) stimulates SOD and catalase activities (Hamdi et al., 2011). The present study shows that, in the same range of concentrations, ODN induces a dose-dependent increase of SOD and catalase mRNA levels in cultured astroglial cells, indicating that ODN regulates not only enzyme activity but also gene transcription. Although SOD and catalase genes exhibit hallmarks of typical housekeeping genes, it has been shown that their promoters encompass consensus sequences for regulatory elements such as metal-responsive element, antioxidant responsive element, glucocorticoid-response element, and nuclear factor-κB (Nenoi et al., 2001; Zhu et al., 2001; Zelko et al., 2002), suggesting that these genes are actually regulated in the CNS. As a matter of fact, SOD and catalase gene expression is selectively increased by inflammatory mediators such as interleukin-1β, interferon (IFN)-γ, IFN-β, or lipopolysaccharides in astrocytes (Mokuno et al., 1994; Kifle et al., 1996; Vergara et al., 2010). Previous data have shown that ODN is specifically produced by astroglial cells (Tonon et al., 1990; Malagon et al., 1993; Compère et al., 2010) and that its release is regulated by various factors including agonists of formyl peptide receptors (Tokay et al., 2008) which are involved in inflammation. These observations suggest that ODN may act as an autocrine factor to finely regulate SOD and catalase gene expression in the brain.

Kinetic experiments indicate that the action of ODN on antioxidant enzyme gene transcription is very rapid but transient. Nevertheless, ODN exerts a protective effect against H_2_O_2_-reduced SOD and catalase mRNA levels. Similar time-response curves have already been observed on SOD and catalase activities, in cultured astrocytes (Hamdi et al., 2011). These data suggest that ODN-induced rapid activation of antioxidant systems is required for the long-lasting inhibition of the deleterious effect of H_2_O_2_. That ODN-induced increase of transcription and activity of antioxidant enzymes is responsible, at least in part, for inhibition of cell death is consistent with previous data showing that SOD and catalase blockers suppress the protective effect of ODN against H_2_O_2_-induced astrocyte apoptosis (Hamdi et al., 2011). Furthermore, it has been reported that, in cultured astrocytes, overexpression of SOD is able to prevent ROS-induced alteration of mitochondrial integrity, caspase-3 activation and thus cells apoptosis (Yang et al., 2008).

Previous studies have shown that, in cultured astrocytes, ODN can interact with either CBR associated with the GABA_A_ receptor (Gandolfo et al., 1999) or with a G protein-coupled receptor positively coupled to PLC (Patte et al., 1995; Leprince et al., 2001). Here, we found that the inhibitory effects of ODN on H_2_O_2_-evoked reduction of SOD and catalase mRNA levels and activities were suppressed by the ODN analog cyclo_1-8_[DLeu^5^]OP but were not affected by the specific CBR antagonist flumazenil. It has been reported that the cyclic analog of ODN exerts potent antagonistic activities on ODN-induced polyphosphoinositide turnover increase and intracellular calcium mobilization in rat astrocytes (Leprince et al., 2001). Thus, these data indicate that the antioxidant action of ODN is mediated through the activation of the G protein-coupled receptor.

We next investigated the signaling cascade involved in the effect of ODN on endogenous antioxidant systems. ODN blockage of H_2_O_2_-evoked inhibition of SOD and catalase gene transcription and enzyme activities was totally abrogated by the PKA inhibitor H89, while the PKC inhibitor chelerythrine had no effect. That, the ODN G protein-coupled receptor could stimulate the AC/PKA transduction cascade is in agreement with recent data indicating that ODN increases the production of cAMP in astrocytes (Hamdi et al., 2012). Altogether, these observations indicate that the antioxidant action of ODN against H_2_O_2_-induced oxidative stress can be specifically ascribed to the activation of the AC/PKA signaling pathway. Consistent with this notion, it has been shown that the SOD and catalase promoters contain a cAMP-responsive element-like sequence (Das et al., 1995; Zelko et al., 2002; Colombo and Moncada, 2009) and that siRNA knockdown of cAMP-responsive element-binding protein (CREB) or inhibition of CREB phosphorylation blocks the expression of SOD in the rat hypothalamus (Hsieh et al., 2008) and the expression of catalase in human vascular endothelial cells (Colombo and Moncada, 2009), respectively. The fact that ODN provokes ERK phosphorylation via a cAMP-dependent pathway in astrocytes (Hamdi et al., 2012), strongly suggests that the stimulatory effect of ODN on SOD and catalase expression can also be ascribed to activation of the ERK-type MAP kinase transduction pathway.

The protective effect of ODN against H_2_O_2_-reduced antioxidant enzyme expression and activities might have a physiopathological significance in neurodegenerative diseases and stroke. CNS is sensitive to oxidative stress due to its high metabolic rate and high levels of unsaturated lipids so that up-regulation of antioxidant enzyme systems in astroglial cells could be beneficial against cell death observed during and after ischemia and neurodegenerative diseases. In agreement with this hypothesis, we have recently shown that the endozepine ODN exerts a potent protective action against apoptosis induced by oxidative stress in astrocytes (Hamdi et al., 2011, 2012) and that the anti-apoptotic effect of ODN is attributable to activation of the antioxidant enzymes that act as scavengers of H_2_O_2_ and ROS (Hamdi et al., 2011). The fact that the glioprotective action of ODN is likely mediated through the metabotropic receptor is of particular interest. Previous data indicate that ODN induces a wide range of activities through activation of CBR (Tonon et al., 2006). In particular, ODN has been initially described as an anxiogenic peptide (De Mateos-Verchere et al., 1998). Since cyclic analog of ODN do not recognize CBR (Leprince et al., 2001), the development of specific cyclic agonists that would selectively mimic the glioprotective effect of ODN might prove useful for the treatment of ischemia and neurodegenerative diseases.

In conclusion, the present study has demonstrated that the endozepine ODN, acting through a metabotropic receptor sensitive to the cyclo_1-8_[DLeu^5^]OP antagonist, exerts a potent antioxidant action against H_2_O_2_-induced oxidative stress in astrocytes. This antioxidant effect of ODN is attributable to activation of both gene expression and activities of enzymatic antioxidant systems and can be ascribed to the stimulation of the AC/PKA transduction pathway.

## Conflict of Interest Statement

The authors declare that the research was conducted in the absence of any commercial or financial relationships that could be construed as a potential conflict of interest.

## Abbreviations

AC: adenylyl cyclase
CBR: central-type benzodiazepine receptors
CREB: cAMP-responsive element-binding protein
DBI: diazepam-binding inhibitor
GADPH: glyceraldehyde-3-phosphate dehydrogenase
H2O2: hydrogen peroxide
ODN: octadecaneuropeptide
PBS: phosphate buffered saline
PKA: protein kinase A
PKC: protein kinase C
PLC: phospholipase C
ROS: reactive oxygen species
SOD: superoxide dismutase.
